# DNA methylation estimates of immune cell abundance have prognostic potential in triple negative breast cancer

**DOI:** 10.1186/s13148-026-02052-w

**Published:** 2026-01-27

**Authors:** Sarah Williams, Darren Korbie, Matt Trau, Kelly A. Avery-Kiejda, Rodney J. Scott, Braydon Meyer, Melissa C. Southey, Roger L. Milne, Pierre-Antoine Dugué, Susan J. Clark, Clare Stirzaker, Ruth Pidsley

**Affiliations:** 1 Cancer Ecosystems Program, Epigenetics Research Laboratory, Garvan Institute of Medical Research, 384 Victoria Street, Darlinghurst, Sydney, NSW 2010 Australia; 2 School of Clinical Medicine, UNSW Medicine and Health, Sydney, NSW 2010 Australia; 3 Centre for Personalised Nanomedicine, The University of Queensland, St Lucia, QLD 4072 Australia; 4 Molecular Medicine, NSW Health Pathology, New Lambton Heights, NSW 2305 Australia; 5 Cancer Detection & Therapy Research Program, Hunter Medical Research Institute, Newcastle, NSW 2305 Australia; 6 Discipline of Medical Genetics, School of Biomedical Sciences and Pharmacy, University of Newcastle, Callaghan, NSW 2308 Australia; 7 Precision Medicine, School of Clinical Sciences at Monash Health, Monash University, Clayton, 246 Clayton Road, Clayton, VIC 3168 Australia; 8 Cancer Epidemiology Division, Cancer Council Victoria, Melbourne, VIC Australia; 9 Melbourne School of Population and Global Health, The University of Melbourne, Parkville, VIC Australia; 10 Centre for Epidemiology and Biostatistics, Melbourne School of Population and Global Health, The University of Melbourne, Parkville, VIC Australia

**Keywords:** DNA methylation, Epigenetics, Triple negative breast cancer, Immune-cell deconvolution, Prognosis

## Abstract

Immune infiltration is now recognised as an important prognostic factor in triple negative breast cancer (TNBC). DNA methylation, due to its cell-type specificity, offers a promising approach for quantifying immune cell abundance as a biomarker for risk stratification. Here, we used EpiDISH, a DNA methylation-based cellular deconvolution method, to estimate immune cell proportions from genome-wide methylation data across four independent breast cancer datasets. We show that increased methylation-estimated immune cell percentage in TNBC patients is associated with improved outcomes. This highlights the potential of DNA methylation-based estimates of cell composition for prognosis in TNBC.

**Supplementary Information:**

The online version contains supplementary material available at 10.1186/s13148-026-02052-w.

## Introduction

Breast cancer remains the leading cancer diagnosis and cause of cancer-related death in females. Triple negative breast cancer (TNBC) is associated with a higher risk of metastasis and recurrence compared to other breast cancer subtypes [1]. TNBC exhibits a distinct molecular and cellular profile that limits the accuracy of current prognostic tools developed for other breast cancer subtypes, necessitating the development of new clinical tools for risk stratification in TNBC [2].

It is now recognised that tumour-infiltrating lymphocytes (TILs) play an important role in TNBC prognosis [3]. More specifically, greater TIL abundance in early-stage tumours of TNBC and HER2+ subtypes is associated with improved clinical outcomes [4, 5]. Most studies use histological staining, such as hematoxylin and eosin (H&E) to assess TIL presence, however, questions remain around the analytical validity of H&E due to its semi-quantitative nature [6].

DNA methylation status provides an attractive alternative for biomarker development because it is a pervasive modification and more stable than RNA or proteins [7]. DNA methylation status can be detected and quantified from tissue, including fresh frozen or formalin fixed paraffin embedded tissue (FFPET). In healthy cells, DNA methylation is cell-type specific, meaning DNA methylation profiling can be used to estimate the proportions of different cells in a sample, including immune cells.

EpiDISH [8] is an established and highly utilised bioinformatic tool for the cellular deconvolution of DNA methylation data, developed using epigenetic data from a range of samples including whole blood, cell lines and tissue samples, including breast tissue [9]. It has since been widely applied for the quantification of cell-type composition in diverse contexts [10, 11], including in studies on breast cancer tissue [12, 13]. Importantly, in a benchmarking study evaluating 16 DNA methylome deconvolution algorithms, EpiDISH was identified as the overall best performing algorithm [14].

In this study we sought to assess the value of the novel approach of using DNA methylation-based estimates of immune cell proportions for breast cancer prognosis, leveraging our unique in-house TNBC datasets, with long term follow up, as well as well-characterised public datasets. Our in-house datasets include the Melbourne Collaborative Cohort Study (MCCS) which is a large population-based cohort [15] and the National Breast Cancer Foundation (NBCF) curated cohort, enriched for events; neither methylation cohort has been previously studied for association between cellular composition and disease prognosis, and the NBCF data has not been previously published. In conjunction with publicly available TNBC datasets, we identified an association between DNA methylation-based estimates of immune cell fraction and outcome in four independent TNBC cohorts. Importantly, we also report an association between H&E-based and DNA methylation-based estimates of immune cell fraction.

## Material and methods

### Ethics approval

The study protocol was approved by the Hunter New England Research Ethics Committee (NSW HREC Reference No: HREC/09/HNE/153), Newcastle, New South Wales and Princess Alexandra Hospital Human Research Ethics Committee (Research Protocol: 2007/165) Brisbane, Queensland. Ethics approval (HREC 2019_ETH12651) was obtained from St Vincent’s Hospital (Sydney) HREC.

### Clinical cohort datasets

MCCS: Illumina HumanMethylation 450K (HM450K) DNA methylation array data from FFPET (n = 419) was obtained from the Melbourne Collaborative Cohort Study [15] and includes samples of TNBC (n = 63), HER2+ (n = 29), Luminal A (n = 236) and Luminal B (n = 86) subtypes.

NBCF: TNBC samples (n = 62) were obtained as FFPET blocks for HM450K DNA methylation profiling from archives of NSW Health Pathology, John Hunter Hospital, Newcastle, Australia; University of Queensland and Garvan Institute of Medical Research, together with clinical data.

GSE141441: Processed HM450K DNA methylation array data and accompanying clinical data for TNBC patients (n = 166) was downloaded from the GEO repository GSE141441 [16].

TCGA: The Cancer Genome Atlas (TCGA) HM450K DNA methylation array data was downloaded from NCI Genomics Data Commons Portal in 2018. Clinical data (clinical biospecimen files) was obtained from cBioPortal (TCGA firehouse legacy) in 2025. 67 of 118 TNBC cases were selected for download due to their overlap with samples from a previous study in which TIL estimates were scored by a pathologist from H&E images [17].

See Additional File 1, Table 1 and Table S1 for further patient details.

**Table 1:** Cohort details of triple negative breast cancer (TNBC) patient sample

	MCCS (n = 63)	NBCF (n = 62)	GSE141441 (n = 166)	TCGA (n = 67)
Age, Years				
Mean (range)	61.3 (47.3–79.9)	54.4 (32–88)	59.1 (32–92)	55.7 (29–90)
Grade				
1	2 (3.2%)	2 (3.2%)	2 (1.2%)	0
2	10 (15.9%)	3 (4.8%)	25 (15.1%)	0
3	45 (71.4%)	57 (91.9%)	121 (72.9%)	0
NA	6 (9.5%)	0	18 (10.8%)	67 (100%)
T stage				
T1	41 (65.1%)	9 (14.5%)	0	21 (31.3%)
T2	20 (31.7%)	18 (29.0%)	0	38 (56.7%)
T3	0	3 (4.8%)	0	6 (9.0%)
T4	1 (1.6%)	1 (1.6%)	0	2 (3.0%)
NA	1 (1.6%)	31 (50.0%)	166 (100%)	0
N stage				
N0	44 (69.8%)	16 (25.8%)	0	39 (58.2%)
N1	9 (14.3%)	11 (17.7%)	0	17 (25.4%)
N2	3 (4.8%)	3 (4.8%)	0	7 (10.5%)
N3	4 (6.3%)	1 (1.6%)	0	4 (6.0%)
NX	3 (4.8%)	1 (1.6%)	0	0
NA	0	30 (48.4%)	166 (100%)	0
M stage				
M0	46 (73.0%)	10 (16.1%)	0	52 (77.6%)
M1	3 (4.8%)	0	0	1 (1.5%)
MX	14 (22.2%)	5 (8.1%)	0	14 (20.9%)
NA	0	47 (75.8%)	166 (100%)	0
Follow up time, months				
Mean (range)	202.3 (13.2–356.4)	58.3 (7–194)	90.5 (6.9–330.5)	36.5 (0.2 –109.8)
Survival state*				
Events	13 (20.6%)	30 (48.4%)	69 (41.6%)	11 (16.4%)

Clinicopathological characteristics of the TNBC patients in the MCCS, NBCF, GSE141441 and TCGA datasets. Table includes patients whose tumour samples passed quality control during DNA methylation data preprocessing.

*Event is defined as disease-specific survival (DSS) for the MCCS and NBCF datasets, disease-free survival (DFS) in the GSE141441 dataset and overall survival (OS) for the TCGA dataset. NA = Not Available

### DNA methylation profiling

NBCF DNA methylation status was quantified using Illumina HumanMethylation 450K BeadChip following manufacturer’s instructions. All raw intensity data (IDAT) files were imported into the R environment (v4.3.1) using package *minfi* (v1.48.0) (see Additional File 1 for further details on preprocessing).

### Statistical analysis

For initial data visualisation, we used a multi-dimensional scaling (MDS) plot on the 1,000 most variable probes in the MCCS dataset, using *mdsPlot* function (*minfi* package, v1.48.0). To estimate cellular composition, we used R package *EpiDISH* (v2.18.0) [8], using the centEpiFibIC.m reference dataset. Wilcoxon rank sum tests were employed to compare cellular percentages between breast cancer subtypes; results were corrected for multiple comparisons with False Discovery Rate (FDR) and statistical significance set at *p* < 0.05. Principal Component Analysis (PCA) was performed using R package *stats* (v4.3.1) and the top 5 principal components selected for further analysis. Associations with clinical and technical variables were assessed using linear regression models.

### Survival analysis

Survival analysis was implemented in R package *survival* (v3.7-0) using disease-specific survival (DSS) as primary endpoint for the MCCS (71 events, of which 13 TNBC) and NBCF (30 events) datasets, disease-free survival (DFS) in the GSE141441 cohort (69 events), and overall survival (OS) for the TCGA dataset (11 events). Cox proportional hazards (CPH) model (Hazard Ratio (HR) and 95% Confidence Interval (CI) reported throughout) and log-rank tests were used to examine associations between DNA methylation-based cell fractions and survival, and visualised using Kaplan–Meier plots. Package details and additional methods for all bioinformatic analyses can be found in Additional File 1.

## Results

To investigate the prognostic utility of DNA methylation-based estimates of cellular composition in TNBC, and in other breast cancer subtypes for comparison, we first performed a genome-wide DNA methylation analysis of primary breast cancer FFPET samples from two independent HM450K array datasets. The first, MCCS, comprised 419 women of which 63 had been diagnosed with TNBC, 29 with HER2+, 236 with Luminal A, 86 with Luminal B, and 5 had unknown subtype (Table 1, Table S1). The second cohort, NBCF, was curated from 62 TNBC patients from three institutions (Table 1) and the methylation data generated as part of this study has been made publicly available on the NCI GEO database (GSE308164) (www.ncbi.nlm.nih.gov/geo).

**Fig. 1:**
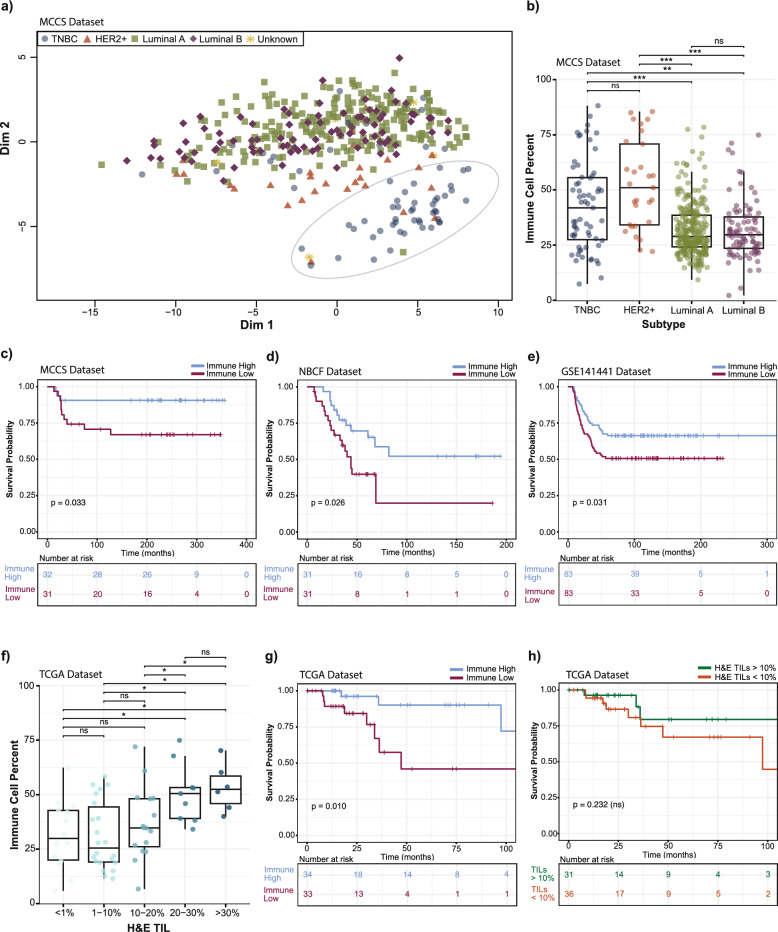
Cellular deconvolution of the triple negative breast cancer (TNBC) methylome reveals association between methylation-estimated immune percentage and prognosis in four independent datasets. **a-b** Exploratory analysis of MCCS breast tumour methylation data. Multidimensional Scaling (MDS) plot (**a**) of the 1000 most variable methylation CpG sites shows clustering by breast cancer subtype. TNBC (blue) cluster separately from Luminal A (green) and Luminal B (purple). HER2+ samples (orange) cluster intermediate between TNBC and Luminal subtypes. Circle indicates the TNBC cluster. Boxplots (**b)** of EpiDISH estimated immune cell percentage * *p* < 0.05, ** *p* ≤ 0.01, *** *p* ≤ 0.001, Wilcoxon-rank sum test. **c-e** Kaplan–Meier plots of relationship between survival and immune cell percentage, with p-values obtained from log-rank tests. EpiDISH estimated immune cell percentage is associated with **c** disease specific survival (DSS) in the MCCS TNBC samples, **d** DSS in the TNBC NBCF samples and **e** disease free survival (DFS) in the GSE141441 dataset. **f** Correlation between H&E estimated Tumour Infiltrating Lymphocyte (TILs) and EpiDISH estimated immune cell percentage demonstrates concordance between methods * *p* < 0.05, ** *p* ≤ 0.01, *** *p* ≤ 0.001, Wilcoxon-rank sum test**. g-h** Kaplan–Meier plots of relationship between overall survival and **g** EpiDISH estimated immune cell percentage and **h** H&E scored TIL percentage in the TCGA dataset. Blue line: ‘immune high’, maroon line: ‘immune low’, binarized by median DNA methylation-estimated immune cell percentage for each breast subtype within each cohort. H&E TILs above (green) and below (orange) the median TILs (10%)

Initial characterisation of the MCCS methylation data with an MDS plot showed that DNA methylation clusters samples according to breast cancer subtype (Fig. 1a**)**. Most notably, TNBCs form a distinct cluster apart from other breast cancers. HER2+ samples are positioned intermediate between TNBC and Luminal subtypes, and Luminal A and B subtypes are indistinguishable from one another. Subsequent PCA of the MCCS methylation data, in comparison with clinical data, showed that breast cancer subtype was the most strongly associated clinical variable with PC1 (ANOVA, *p* = 1.80 × 10^–23^) (Table S2a). The methylation-based clustering by breast cancer subtype was expected given both the known cellular differences between subtypes [3] and the known cell-type specificity of DNA methylation. To test whether the methylation differences between tumour subtypes were indeed related to differences in cellular composition, we employed the highly regarded methylation-based cellular deconvolution method EpiDISH [8] to estimate epithelial, fibroblast and immune cell percentage for each sample (Table S3). Analysis of EpiDISH methylation-estimated cell percentages with the same PCs showed that PC1 was even more significantly associated with methylation-estimated immune cell content (linear regression, *p*  = 1.61 × 10^–30^) than with breast cancer subtype (Figure S1, Table S2b). Comparison between subtypes showed that TNBC has a greater percentage of immune cells compared to luminal cancers: TNBC (median = 41.9%) compared to 1) Luminal A (median = 28.9%) (Wilcoxon rank sum test, *p* < 0.001) and 2) Luminal B (median = 29.7%) (Wilcoxon rank sum test, *p* = 0.002) (Fig. 1b, Figure S2, Table S3). HER2+ exhibited a comparable immune cell percentage to TNBC (median = 51.0%) (Fig. 1b). Next, we applied EpiDISH to the NBCF TNBC samples, which showed that these samples had comparable immune cell percentages to the MCCS TNBC samples (Figure S3, Table S4).

Given the established role of TILs in TNBC prognosis [4, 5] we were interested to explore the variability of methylation-estimated immune cell content in our TNBC sample set and its prognostic utility. We therefore conducted PCA independently on only TNBC samples from the NBCF and MCCS datasets and identified that immune cell percentage was strongly associated with PC2 in NBCF (linear regression, *p* = 6.41 × 10^–22^) (Figure S4, Table S2c) and both PC1 (linear regression, p = 1.26 × 10^–06^) and PC2 (linear regression, *p* = 5.77 × 10^–11^) in MCCS TNBC samples (Figure S5, Table S2d). Next, we evaluated the association between immune cell percentage and DSS using Cox proportional hazards, and log rank test with immune cell percentage binarised on the median, splitting patients into ‘immune high’ or ‘immune low’. In TNBC samples from both MCCS and NBCF datasets, a higher immune cell percentage was associated with longer DSS (log rank test, MCCS: *p* = 0.033, NBCF: *p* = 0.026) (Fig. 1c and d, Table S5). Immune cell percentage as a continuous variable was also associated with survival in the NBCF cohort (CPH, *p* = 0.019, HR (95% CI) = 0.98 (0.96–0.996)), and remained significant after adjusting for age (CPH, *p* = 0.024, HR (95% CI) = 0.98 (0.96–0.997)) and grade (CPH, *p* = 0.022, HR (95% CI) = 0.98 (0.96–0.997)) (Table S6). For comparison, we performed the same analysis on other breast cancer subtypes in the MCCS cohort and found that immune cell percentage was not significantly associated with DSS in the HER2+, Luminal A and Luminal B samples from the MCCS dataset (Figure S6, Table S5, Table S6). We also assessed the relationship between fibroblast and epithelial percentage and DSS in the TNBC samples and found the only significant association was between epithelial percentage and DSS in the NBCF cohort (log-rank test, *p* = 0.049). (Table S5, Table S7).

As further validation, we examined the association between DNA methylation-based immune cell percentage and DFS in a publicly available dataset (GSE141441), comprised of n = 166 TNBC samples [16]. Consistent with the MCCS and NBCF TNBC datasets, a significant association was observed in log-rank analysis between immune cell percentage dichotomised on the median and DFS (log rank test, *p* = 0.031) (Fig. 1e, Table S5). Importantly, this association remained significant after adjusting for age and grade in a Cox model with immune cell percentage as a continuous variable (CPH, *p* = 0.003, HR (95% CI) = 0.98 (0.96–0.99)) (Table S6).

Previous studies using estimated TILs from H&E images had also reported a relationship between TIL abundance and prognosis [4, 5]. To explore the relative utility of EpiDISH measures of methylation-estimated immune cell fraction compared to H&E scored TILs we used a fourth HM450K methylation dataset of TCGA TNBC patients, with matched H&E-scored TILs obtained from *Craven and colleagues* [17] (n = 67) (Table 1). We found a positive relationship between the methylation-estimated immune cell percentage and H&E-scored TIL categories (Fig. 1f, Table S8). We then compared the prognostic utility of methylation-estimated immune cell percentage and H&E scored TILs in the TCGA cohort. First, we used the median methylation-estimated immune cell percentage to dichotomise the cohort as ‘immune high’ (n = 34) and ‘immune low’ (n = 33). Log-rank test showed that higher methylation-estimated immune cell fraction was associated with improved OS (log rank test, *p* = 0.010) (Fig. 1g, Table S5). We then dichotomised the TCGA patients by median pathology-based TIL percentage of 10% (as also used in [18]), giving two groups comprising > 10% TILs (n = 31) and < 10% TILs (n = 36). Log-rank test showed no significant differences in OS (log rank test, *p* = 0.232) between these groups (Fig. 1h, Table S5). These results suggest that DNA methylation-based estimates of immune cell percentage may be a more accurate tool for prognostic stratification than H&E scored TILs. Together, our findings consistently demonstrate that immune cell presence estimated by DNA methylation array data is associated with outcome in TNBC.

## Discussion

The prognostic stratification of TNBC patients remains a significant challenge in clinical management. In recent years, TILs have emerged as a promising predictive and/or prognostic biomarker. However, H&E-based TIL scoring is a semi-quantitative measurement that currently lacks accuracy for precise quantitation. DNA methylation is highly cell-type specific, which has led to the development of bioinformatic tools to estimate cellular proportions. Here, we set out to determine if DNA methylation measurements could provide a quantitative measure of TILs for prognostication. Accordingly, we performed genome-wide DNA methylation analysis of four independent TNBC datasets to test for an association between methylation-estimated immune cell content and patient outcome in TNBC alone, and in relation to other breast cancer subtypes.

We observed that the methylome of TNBC samples was distinct from the other subtypes, in line with previous studies [19]. We also found that DNA methylation-estimated immune cell percentages were higher in both TNBC and HER2+ tumours compared to Luminal cancers, as previously reported from histopathology studies [3]. Importantly, we found a significant association between higher methylation-estimated immune cell presence and improved prognosis within all four independent TNBC datasets. This association was retained after adjustment for covariates age and grade, in both the NBCF and GSE141441 cohorts where there was an appropriate number of events and available clinical information. Interestingly, we also observed a trend towards increased methylation-estimated immune cell presence and improved prognosis in HER2+ tumours. Although limited by HER2+ sample numbers in the current study, this trend is in line with H&E findings that TIL presence is associated with positive clinical outcome in HER2+ tumours [5].

Our results are in agreement with a recent DNA methylation study by Aine and colleagues [20], in which tumours with high immune cell infiltration (subtype ‘Basal3’ defined by tumour methylome profiles and gene expression) were associated with better patient survival than other TNBC tumour subtypes. In further agreement, Craven et al. [17] previously applied a deconvolution method (CIBERSORT) on gene expression data to quantify TILs in the same subset of TCGA patient tumours used in the current study. As in our study, CIBERSORT deconvolution identified an association with H&E TIL estimates, as well as associations with patient prognosis.

There are a number of different methods available for tumour microenvironment (TME) deconvolution including CIBERSORT, BayesPrism and MuSiC for transcriptomic data [21] and EpiDISH, Meth atlas and MethylResolver for DNA methylation data [14]. In addition, bioinformatic tools such as hierarchical EpiDISH (HepiDISH) [22] and HiTIMED [23] can subdivide the immune cell fraction further into immune cell subtypes. Whilst individual immune cell subtypes, including B cells [24] and CD8+ T cells [25], have been associated with prognosis in TNBC we focused our analysis on overall immune cell abundance as a more robust measure given our moderate cohort sizes. Further work will be required to determine the optimum molecular data type and bioinformatic tool for TME deconvolution for prognostic stratification in TNBC.

Studies have shown that measurement of TILs also have predictive value for treatment decisions in TNBC [6]. H&E scored TILs and Programmed Death-Ligand 1 (PD-L1) expression are currently used to identify TNBC tumours likely to respond to immunotherapy [26]. As such, DNA methylation estimates of immune cell percentage may provide a complementary approach for identifying breast cancers with potential to respond to immunotherapy.

A limitation of our study is the lack of clinical and molecular data associated with the cohorts which restricted our analyses. For example, we did not have access to treatment data so were unable to adjust for this in our survival model. However, Loi et al. [4] previously reported that the association of H&E scored TILs and improved patient survival was consistent across different treatment regimens. Previous studies also reported that TILs are associated with outcome in TNBC patients carrying pathogenic germline BRCA1 mutations [27] and that approximately 20% of TNBC patients carry a BRCA1/2 mutation [28]. Whilst we could not examine BRCA1/2 status in our cohorts, the interaction between BRCA1/2 mutations and immune infiltration remains an important direction for further investigation.

For the purposes of discovery we used cohort-specific median immune cell percentage as a pre-defined cutoff to dichotomise patients into high and low immune groups. In the next stage of this work an absolute threshold will need to be established for clinical use. Notably, immune cell percentage as a continuous variable was associated with outcome in only two cohorts (NBCF and GSE141441). This is likely due to the higher number of events in these cohorts, which provided greater statistical power to detect an association (noting that the NBCF was a curated cohort, and GSE141441 was the largest TNBC cohort). Therefore, larger studies of TNBC patients with greater event rates will be essential to further validate the relationship between immune cell proportion and survival measures with adjustment for relevant clinical covariates.

Overall, our study demonstrates the potential utility of DNA methylation as a molecular tool to estimate immune cell abundance for prognostic stratification in TNBC. Our findings highlight the positive association between H&E based TIL percentages and EpiDISH methylation-estimated immune cell percentage. The next step is to harness these results to develop a targeted methylation test to capture immune cell abundance and apply to larger cohorts to validate its prognostic and predictive utility for treatment selection.

## Supplementary Information

Additional file1 (DOCX 46 kb)

Additional file2 (PDF 4892 kb)

Additional file3 (XLSX 36 kb)

## Data Availability

NBCF data generated and analysed during the current study are publicly available at NCBI GEO (www.ncbi.nlm.nih.gov/geo) under accession number GSE308164. MCCS data is available upon request to PEDIGREE https://www.cancervic.org.au/research/epidemiology/pedigree.

## Abbreviations

CI: Confidence interval
CPH: Cox proportional hazards
DFS: Disease-free survival
DSS: Disease specific survival
FDR: False discovery rate
FFPET: Formalin fixed paraffin embedded tissue
H&E: Hematoxylin and eosin
HR: Hazard ratio
HREC: Human research ethics committee
IDAT: Intensity data
MCCS: Melbourne collaborative cohort study
MDS: Multi-dimensional scaling
NBCF: National breast cancer foundation
OS: Overall survival
PCA: Principal component analysis
PD-L1: Programmed death-ligand 1
TCGA: The cancer genome atlas
TILs: Tumour infiltrating lymphocytes
TME: Tumour microenvironment
TNBC: Triple negative breast cancer
