# Supplementary material for: DNA methylation estimates of immune cell abundance have prognostic potential in triple negative breast cancer
Source: Clin Epigenetics. 2026 Jan 27;18:128. doi: 10.1186/s13148-026-02052-w (PMC13330045; doi:10.1186/s13148-026-02052-w)
Supplement: Supplementary file 1 — Additional file1 (DOCX 46 kb) [file 13148_2026_2052_MOESM1_ESM.docx]

**Supplemental Information**

**Extended Methods**

**Clinical samples**

***MCCS***

The samples included in this study were obtained from the Melbourne Collaborative Cohort Study (MCCS) [1]. 41,513 participants were recruited between 1990 and 1994. A subset of incident breast tumours were stored as formalin-fixed paraffin-embedded tissues (FFPET) at 4°C for 15 years. Deaths due to breast cancer to 30^th^ April 2023 were ascertained by linkage to the Victorian Registry of Births, Deaths and Marriages. Survival time from breast cancer diagnosis to death, based on the International Classification of Diseases (ICD-10) code C509, or end of follow up. Immunohistochemical staining and breast cancer subtyping was conducted using the methods outlined by Blows et al [2]. In brief, luminal tumours were those with positive staining for the estrogen receptor (ER) or progesterone receptor (PR). Luminal A tumours were classified as being Human Epidermal Growth Factor Recepter 2 (HER2) negative, whilst Luminal B tumours were HER2+. The non-luminal tumours were subdivided into HER2+ tumours and HER2- tumours, i.e. Triple Negative Breast Cancer (TNBC) tumours. The clinical characteristics of the patients used in the current study are summarised in **Table 1 and Table S1**.

***NBCF***

Samples were obtained as FFPET blocks from archives of the Hunter Area Pathology Service, John Hunter Hospital, Newcastle, Australia, the University of Queensland and the Garvan Institute of Medical Research. Samples were collected between 1991 and 2012. Clinical characteristics of patients used in the current study are shown in **Table 1**.

**DNA methylation arrays**

From the NBCF patients, DNA was extracted from FFPET with the Gentra Puregene Tissue Kit (Qiagen) as previously described [3]. DNA was treated with sodium bisulphite using the EZ-96 DNA methylation kit (Zymo Research). DNA methylation was quantified using the Illumina HumanMethylation 450K BeadChip (HM450K) (Illumina) following the manufacturer’s instructions. Raw DNA methylation data for the MCCS dataset was obtained from MCCS, with data generated as previously described [4].

For each cohort raw intensity data (IDAT) files were imported into the R environment (version 4.3.1) using the package *minfi* (version 1.48.0) [5]. Data quality of samples was checked with plots of signal from control probes on the array. 16 MCCS samples were removed due to poor detection p-value leaving n=419 MCCS samples and all n=62 NBCF samples. Data was then normalised within each dataset with *preprocessFunnorm.* Poor quality probes with a detection P value > 0.05 in at least 10% of samples were removed. Probes mapping to overlapping SNPs or multiple locations were removed to reduce the chance of technical artefacts [6]. β values were calculated from the unmethylated (U) and methylated (M) signal [M/( U + M + 100)] and ranged from 0 to 1 (0 to 100% methylation).

**Publicly available genome-wide DNA methylation and H&E data**

***GSE141441 dataset****:*

Processed HM450K array methylation data was downloaded from the GEO repository GSE141441 [7] along with matched clinical characteristics.

***TCGA dataset:***

HM450K array methylation datasets were downloaded from The Cancer Genome Atlas (TCGA) from the NCI Genomic Data Commons portal in 2018. Methylation data was processed as outlined in Lu et al. 2020 [8]. Clinical data (clinical biospecimen files) was obtained from cBioPortal (TCGA, firehouse legacy) downloaded on the 28^th^ February 2025. Of the 713 unique samples with available HM450K array data, we selected 118 TNBC samples, defined as having “Negative” ER Status (‘ER Status By IHC), “Negative” PR Status (‘PR status by ihc’) as well as either: “Negative” HER2 fluorescence in situ hybridization (FISH) result (‘HER2 fish status’) and “Negative”, “Equivocal” or “Indeterminate” HER2 assessed by immunohistochemistry (IHC) (‘IHC-HER2’) or “Negative”, “Equivocal” or “Indeterminate” HER2 assessed by IHC (‘IHC-HER2’) only. Of these 118 TNBC cases, 67 overlapped with samples from a study by Craven and colleagues [9] with Tumour Infiltrating Lymphocyte (TIL) estimates scored by a pathologist from hematoxylin & eosin (H&E) images, and were used for comparative analysis.

**DNA methylation analysis**

***Disease subtype analysis***

For initial data visualisation, we used a multi-dimensional scaling (MDS) plot of the 1000 most variable CpG sites in the MCCS dataset, using the *mdsPlot* function (*minfi* package, version 1.48.0).

***Cellular deconvolution of methylation data***

To estimate cellular composition, we used the R package EpiDISH [10,11] (version 2.18.0) using the centEpiFibIC.m reference datasets and ‘RPC’ method. The RPC method was selected as a methodological study found it to be a robust inference framework for a range of tissue types and for the known noise levels in methylation microarray data [10]. Wilcoxon rank sum tests were used to compare cellular percentages between breast cancer subtypes, results were corrected for multiple comparisons using the FDR, with significance set at p < 0.05. The R package *ggpubr* (version 0.6.0) was used to visualise these comparisons as boxplots with overlaid points.

***Principal Component Analysis***

Principal Component Analysis (PCA) was conducted using the R package *stats* (version 4.3.1). To investigate the associations between principal components and clinical and technical variables in the MCCS cohort, we selected the top 5 principal components (PC) for further analysis. Variables included both categorical (Sentrix ID, Sentrix Position, breast cancer subtype and mortality status) and continuous variables (DNA methylation-based immune cell percentage, epithelial percentage and fibroblast percentage, and age at diagnosis). For each of the above variables, a linear model was fit with each of the top 5 PCs as the outcome. For continuous variables, the p-value was taken from the regression and the corresponding t-statistics was extracted. For categorical variables the p-value was derived from ANOVA of the model and the associated F-statistics was recorded. All p values were corrected for multiple comparisons using the FDR with significance set at p < 0.05. Principal component scores were visualised using scatter plots of PC1 versus PC2 using the *ggplot2* package (version 3.5.1).

The same analysis was conducted for the TNBC only samples in the NBCF and MCCS datasets, with the exception of breast cancer subtype as a variable for analysis.

***Correlation between methylation-estimated immune cell content and H&E scored TILs***

For the 67 TCGA TNBC samples with H&E TIL information, we assessed the correlation between H&E scored TILs and immune cell percentage as estimated via EpiDISH. We first pooled any samples with H&E TILs of 30-40%, 40-50%, 50-60%, 60-70% or >70% into one group of >30% TILs as the number of samples in these categories was small. Wilcoxon rank sum tests were used to compare immune cell percentage as estimated by EpiDISH between H&E TIL categories, results were corrected for multiple comparisons using the FDR, with significance set at p < 0.05. The R package *ggpubr* (version 0.6.0) was used to visualise these comparisons as boxplots with overlaid points.

***Survival analysis***

Survival analysis was implemented in the R package *survival* (version 3.7-0) using survival outcomes: Disease Specific Survival (DSS) (MCSS and NBCF) or Disease-Free Survival (DFS) (GSE141441) and overall survival (OS) (TCGA), as DSS is not available from GSE141441 or TCGA. Patients in each cohort were stratified into ‘immune high’ and ‘immune low’ by median immune cell fraction as estimated by EpiDISH. Log-rank tests were used to examine the association between dichotomised immune cell fraction and visualised using Kaplan-Meier plots, followed by univariate Cox proportional hazards models to extract Hazard Ratios. Next, to assess deconvoluted immune cell fraction as a continuous variable and patient outcome, univariate and multivariate Cox proportional hazards model were used, with the covariates age in all cohorts and grade in the NBCF and GSE141441 cohort to ensure sound statistical analysis by adhering to the rule of 10 events per variable as established by Peduzzi et al [12]. Statistical significance was defined at p < 0.05. The same survival analysis was conducted using epithelial and fibroblast percentage as the variable of interest.

For the TCGA TNBC H&E TIL analysis, samples were binarized by the median TIL% from H&E, consistent with the approach used in analysis of methylation deconvoluted immune cell fraction, creating two groups; > 10% TILs (those in the H&E groups, 10–20%, 20–30%, 30–40%, 40–50%, 50–60%, 60–70%, or > 70%) (n = 31) and < 10% TILs (those in the H&E groups <1% or 1-10%) (n = 36). After applying this dichotomisation threshold, an association with OS was assessed using a log-rank test. Results were visualised with Kaplan-Meier plots and significance was defined at p < 0.05.

**References**

[1] Milne RL, Fletcher AS, MacInnis RJ, et al. Cohort Profile: The Melbourne Collaborative Cohort Study (Health 2020). Int J Epidemiol. 2017 Dec 1;46(6):1757-1757i.

[2] Blows FM, Driver KE, Schmidt MK, et al. Subtyping of breast cancer by immunohistochemistry to investigate a relationship between subtype and short and long term survival: a collaborative analysis of data for 10,159 cases from 12 studies. PLoS Med. 2010 May 25;7(5):e1000279.

[3] Mathe A, Wong-Brown M, Locke WJ, et al. DNA methylation profile of triple negative breast cancer-specific genes comparing lymph node positive patients to lymph node negative patients. Sci Rep. 2016 Sep 27;6:33435.

[4] Wong EM, Joo JE, McLean CA, et al. Tools for translational epigenetic studies involving formalin-fixed paraffin-embedded human tissue: applying the Infinium HumanMethyation450 Beadchip assay to large population-based studies. BMC Res Notes. 2015 Oct 6;8:543.

[5] Aryee MJ, Jaffe AE, Corrada-Bravo H, et al. Minfi: a flexible and comprehensive Bioconductor package for the analysis of Infinium DNA methylation microarrays. Bioinformatics. 2014 May 15;30(10):1363-9.

[6] Chen YA, Lemire M, Choufani S, et al. Discovery of cross-reactive probes and polymorphic CpGs in the Illumina Infinium HumanMethylation450 microarray. Epigenetics. 2013 Feb;8(2):203-9.

[7] Fackler MJ, Cho S, Cope L, et al. DNA methylation markers predict recurrence-free interval in triple-negative breast cancer. NPJ Breast Cancer. 2020;6:3.

[8] Lu J, Wilfred P, Korbie D, et al. Regulation of Canonical Oncogenic Signaling Pathways in Cancer via DNA Methylation. Cancers (Basel). 2020 Oct 30;12(11).

[9] Craven KE, Gokmen-Polar Y, Badve SS. CIBERSORT analysis of TCGA and METABRIC identifies subgroups with better outcomes in triple negative breast cancer. Sci Rep. 2021 Feb 25;11(1):4691.

[10] Teschendorff AE, Breeze CE, Zheng SC, et al. A comparison of reference-based algorithms for correcting cell-type heterogeneity in Epigenome-Wide Association Studies. BMC Bioinformatics. 2017 Feb 13;18(1):105.

[11] Zheng SC, Webster AP, Dong D, et al. A novel cell-type deconvolution algorithm reveals substantial contamination by immune cells in saliva, buccal and cervix. Epigenomics. 2018 Jul;10(7):925-940.

[12] Peduzzi P, Concato J, Kemper E, et al. A simulation study of the number of events per variable in logistic regression analysis. J Clin Epidemiol. 1996 Dec;49(12):1373-9.
