# Supplementary material for: DNA methylation estimates of immune cell abundance have prognostic potential in triple negative breast cancer
Source: Clin Epigenetics. 2026 Jan 27;18:128. doi: 10.1186/s13148-026-02052-w (PMC13330045; doi:10.1186/s13148-026-02052-w)
Supplement: Supplementary file 2 — Additional file2 (PDF 4892 kb) [file 13148_2026_2052_MOESM2_ESM.pdf]

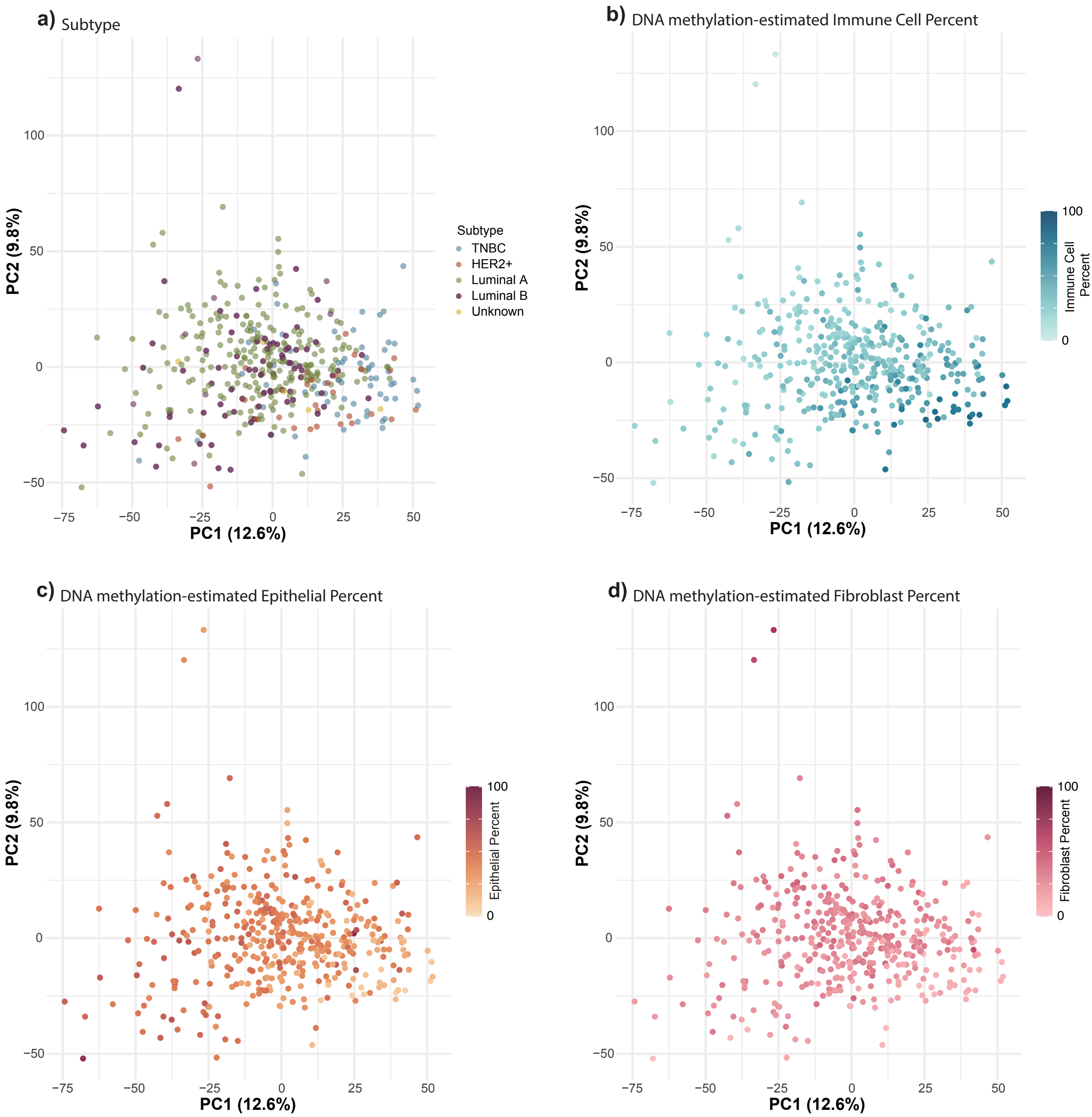

Figure S1. Principal Component Analysis (PCA) of the MCCS dataset reveals association between DNA methylation and DNA methylation-based estimates of cell composition
Plots of PC1 and PC2 from PCA analysis of MCSS TNBC sample DNA methylation coloured by **a.** subtype, **b.** immune cell percentage, **c.** epithelial percentage and **d.** fibroblast percentage.

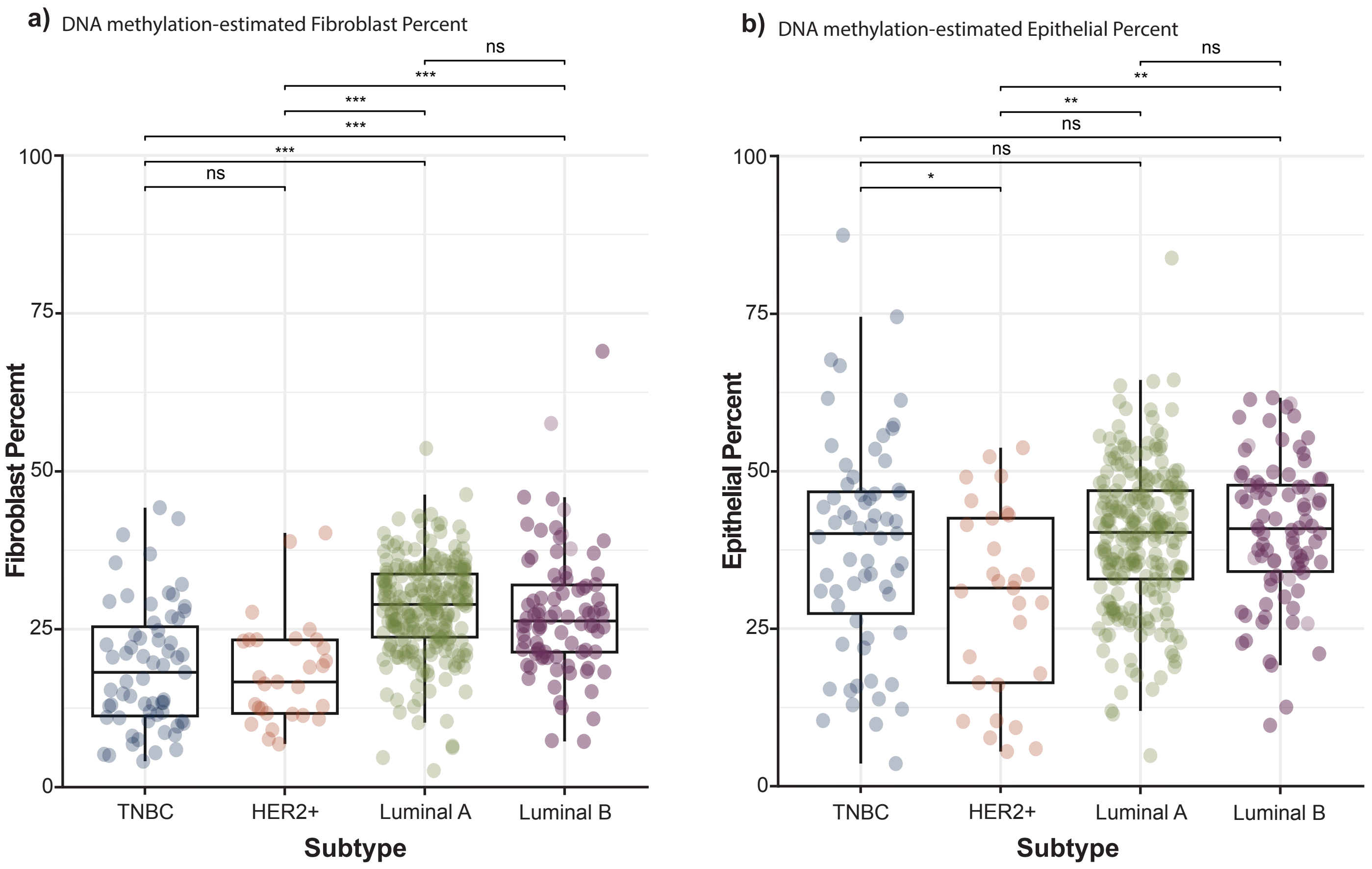

Figure S2. Cellular deconvolution using DNA methylation shows different cellular composition between breast cancer subtypes in the MCCS dataset

EpiDISH estimates of **a**. fibroblasts and **b.** epithelial cell percentage across breast cancer subtypes.* $p < 0.05$, ** $p \leq 0.01$, *** $p \leq 0.001$, Wilcoxon-rank sum test.

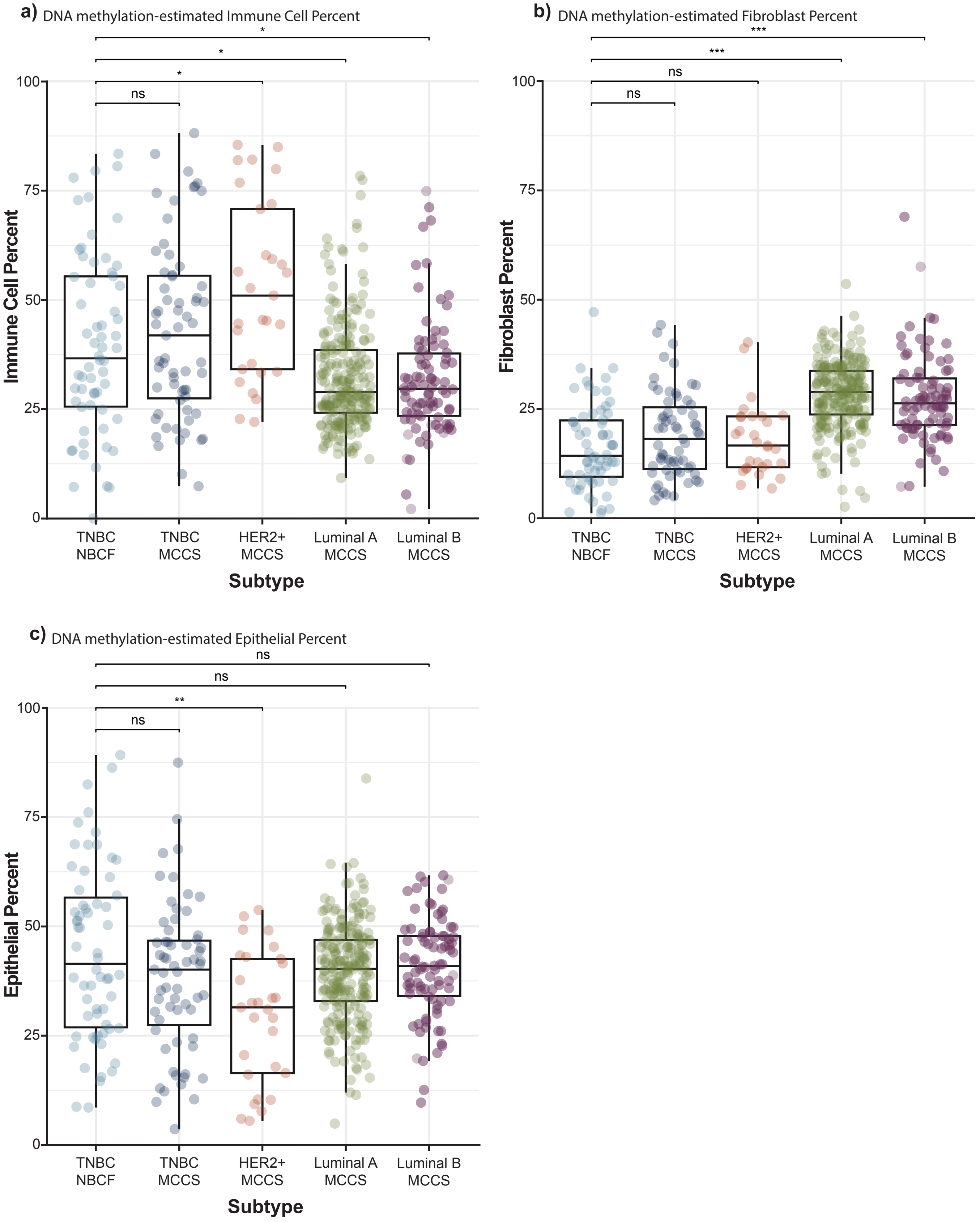

Figure S3. Cellular deconvolution using DNA methylation shows similar cell composition in two TNBC datasets

EpiDISH estimates of cell composition shows the TNBC NBCF and MCCS TNBC samples have similar proportions of **a.** immune cells, **b.** fibroblasts and **c.** epithelial cells. * $p < 0.05$, ** $p \leq 0.01$, *** $p \leq 0.001$, Wilcoxon-rank sum test.

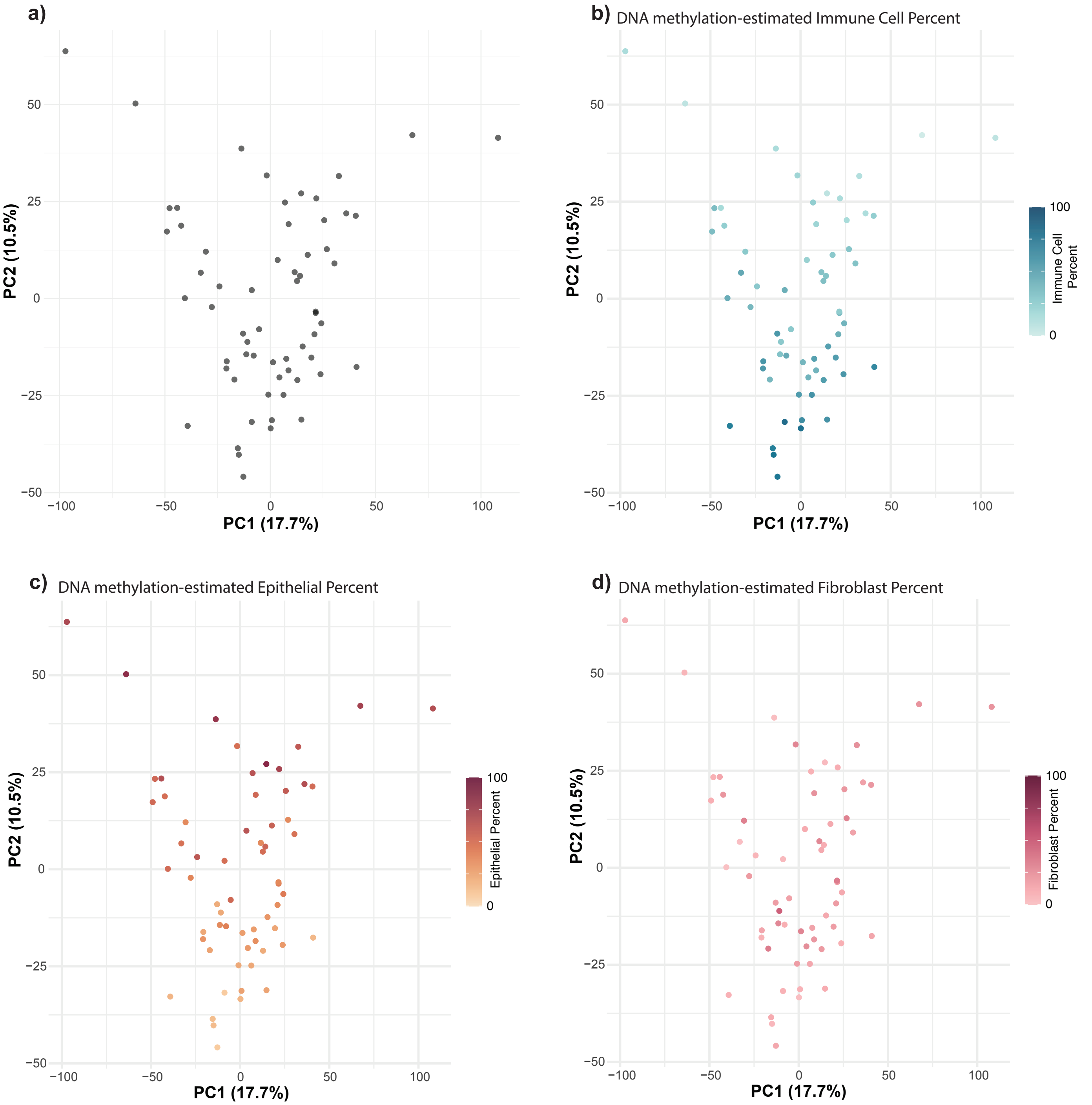

Figure S4. Principal Component Analysis (PCA) of the NBCF dataset reveals cell composition contributes to variance

PCA of the global DNA methylation pattern of tumours in the NBCF dataset. **a-d.** Plots of PC1 and PC2 from PCA analysis of the NBCF DNA methylation dataset (**a**), coloured by percentage of immune cells (**b**), epithelial cells (**c**) and fibroblast cells (**d**).

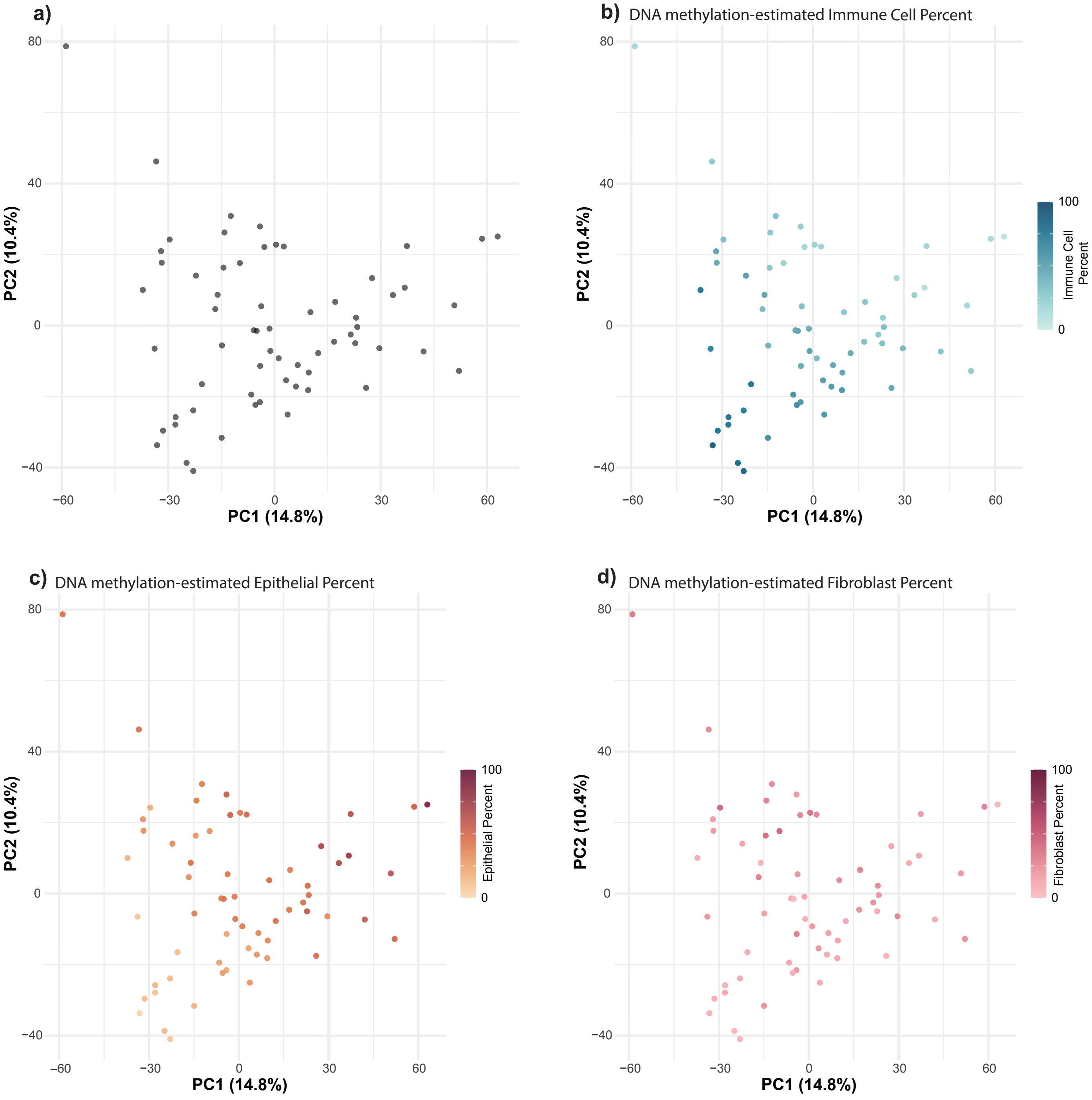

Figure S5. Principal Component Analysis (PCA) of the MCCS TNBC cohort reveals cell composition contributes to variance

PCA of the DNA methylation of MCCS TNBC samples. **a-d**. Plots of PC1 and PC2 from PCA analysis of the MCSS TNBC DNA methylation dataset (**a**), coloured by percentage of immune cells (**b**), epithelial cells (**c**) and fibroblast cells (**d**).

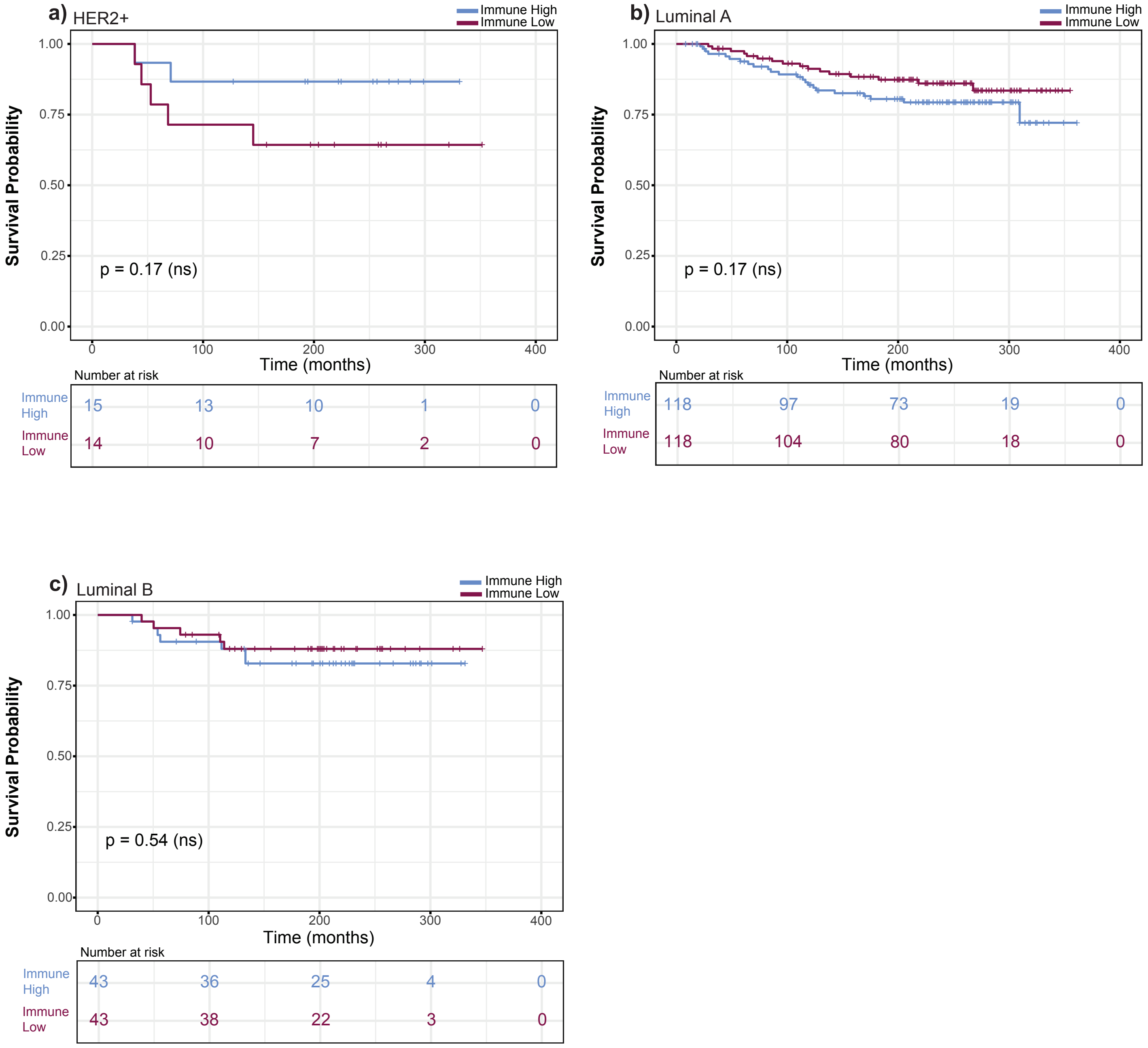

Figure S6. DNA methylation estimates of immune cell percentage are not associated with disease specific survival in non-TNBC subtypes

Kaplan-Meier survival curves with disease specific survival as endpoint in the **a.** HER2+ patients **b.** Luminal A patients and **c.** Luminal B patients of the MCCS dataset. The blue line indicates ‘immune high’ and the maroon line indicates ‘immune low’, defined as samples above or below the median immune cell percentage for the sample subtype within each cohort. p-values obtained from log-rank tests.
